# Gender Differences in Modifying Lumbopelvic Motion during Hip Medial Rotation in People with Low Back Pain

**DOI:** 10.1155/2012/635312

**Published:** 2012-01-23

**Authors:** Shannon L. Hoffman, Molly B. Johnson, Dequan Zou, Linda R. Van Dillen

**Affiliations:** Program in Physical Therapy, Washington University School of Medicine in St. Louis, 4444 Forest Park Boulevard, Campus Box 8502, St. Louis, MO 63108, USA

## Abstract

Reducing increased or early lumbopelvic motion during trunk or limb movements may be an important component of low back pain treatment. The ability to reduce lumbopelvic motion may be influenced by gender. The purpose of the current study was to examine the effect of gender on the ability of people with low back pain to reduce lumbopelvic motion during hip medial rotation following physical therapy treatment. Lumbopelvic rotation and hip rotation before the start of lumbopelvic rotation were assessed pre- and posttreatment for 16 females and 15 males. Both men and women decreased lumbopelvic rotation and completed more hip rotation before the start of lumbopelvic rotation post-treatment compared to pre-treatment. Men demonstrated greater lumbopelvic rotation and completed less hip rotation before the start of lumbopelvic rotation than women both pre- *and* post-treatment. Both men and women reduced lumbopelvic motion relative to their starting values, but, overall, men still demonstrated greater and earlier lumbopelvic motion. These results may have important implications for understanding differences in the evaluation and treatment of men and women with low back pain.

## 1. Introduction

Motor control impairments and lumbopelvic instability have been implicated by many as a cause of low back pain [1–5]. More specifically, several authors suggest that, for some people, low back pain is the result of impaired lumbopelvic motion control [2, 3, 5]. Impaired lumbopelvic motion control may be defined as excessive or early lumbopelvic motion (flexion, extension, rotation, or lateral bending of the lumbar or pelvic regions) during trunk or limb movements. Excessive or early lumbopelvic motion is problematic because as particular trunk or limb movements are performed repeatedly, such as with functional activities, stress may accumulate in specific lumbar or pelvic region tissues and over time may lead to tissue damage and pain [2, 3, 5, 6]. Many studies have demonstrated a relationship between increased or early lumbopelvic motion during trunk or limb movements and low back pain [7–15]. Controlling or limiting lumbopelvic motion during these trunk or limb movements, thereby improving lumbopelvic stability, may be an important component of low back pain treatment [2, 3, 5].

 Lumbopelvic motion during the limb movement of hip rotation has been of particular interest to investigators. Active hip medial and lateral rotation performed in prone are among the limb movements most often associated with an increase in low back pain symptoms [16]. Although hip medial rotation has not been studied, during hip lateral rotation, people with low back pain demonstrate earlier and greater lumbopelvic motion compared to people without low back pain [12]. When lumbopelvic motion is manually restricted during either direction of hip rotation, low back pain symptoms are decreased or eliminated in most people [15]. Limiting lumbopelvic motion during hip rotation, and other movements, is a component of treatment for certain subgroups classified according to the Movement System Impairment model for low back pain [5]. Studies have shown that many people with low back pain can restrict lumbopelvic motion without manual assistance during hip rotation, both within a session [17] and following a 6-week treatment protocol based on the Movement System Impairment model [18].

However, there appear to be factors that affect a person's ability to minimize lumbopelvic motion. People with low back pain may not be able to limit lumbopelvic motion as well as people without low back pain during hip lateral rotation [17]. This may be the result of people with low back pain demonstrating greater [12] and earlier [12, 17] lumbopelvic motion during lower extremity movements than people without low back pain. It is possible that people who display greater impairments in lumbopelvic motion would have a more difficult time limiting lumbopelvic motion with training. Alternatively, proprioceptive deficits identified in people with low back pain may also make it more difficult for them to learn to limit lumbopelvic motion [19, 20]. Within people with low back pain, however, it is unclear what factors may influence the extent to which an individual is able to restrict lumbopelvic motion following treatment.

Previous studies have demonstrated that gender is a factor related to the response to low back pain treatment [21–24]. Gender could also be a factor influencing a person's ability to restrict lumbopelvic motion as prescribed in treatments based on the Movement System Impairment model. Men with low back pain demonstrate less hip medial and more hip lateral rotation range of motion than women with low back pain [25]. Men and women also differ with regard to the amount and timing of lumbopelvic motion observed during hip rotation [26, 27]. During hip lateral rotation, men demonstrate a greater proportion of their total lumbopelvic motion early in the range of hip motion compared to women [27]. During hip medial rotation, men demonstrate significantly greater and earlier lumbopelvic motion than women [26]. It is not clear if these baseline differences between men and women, not only in lumbopelvic motion, but also in hip rotation range of motion, would affect their ability to limit lumbopelvic motion during hip rotation following treatment.

Gender differences in the ability to limit lumbopelvic motion during hip rotation could have important treatment implications. The purpose of this study was to examine the effect of gender on the ability of people with low back pain to improve lumbopelvic movement patterns during hip medial rotation. Improvement was defined as either less lumbopelvic rotation or the completion of more hip medial rotation before the onset of lumbopelvic rotation. Because, in people with back pain, women have been shown to demonstrate less lumbopelvic motion or later lumbopelvic motion during hip medial rotation than men at baseline [26], it may be easier for them to correct these impairments. Therefore, we hypothesized that women would demonstrate greater improvements in these variables following treatment than men.

## 2. Methods

### 2.1. Subjects

Subjects were participants in a randomized controlled clinical trial studying the effects of two physical therapy treatments for low back pain. One of the treatment conditions provided specific treatment based on each individual's low back pain subgroup according to the Movement System Impairment model for low back pain. The other treatment condition provided a general treatment, regardless of low back pain subgroup. Previous research has already suggested that specific treatment results in improvements in lumbopelvic movement patterns, but nonspecific treatment does not [18]. Therefore, subjects for this analysis were selected from only the specific treatment condition to address the question of whether men and women modify their lumbopelvic movement patterns differently. Sixteen male and 16 female subjects were randomly selected from the specific treatment condition for this analysis. Each subject had been classified into either the Rotation or Rotation with Extension subgroup. Individuals classified in either subgroup might be expected to demonstrate lumbopelvic rotation during hip medial rotation.

People with complaints of chronic [28] low back pain for at least 12 months, who were between the ages of 18 and 60 years and were able to stand and walk without assistance, were included in the clinical trial. Low back pain was defined as pain in the region between the twelfth thoracic vertebra and the gluteal fold. People with pain or paresthesia extending into the lower limb, above the knee, were included. Subjects must not have been experiencing an acute flare-up [28] in order to study their typical movement and symptom behaviors. People were excluded from the clinical trial if they had a diagnosis of a spinal deformity including marked kyphosis or scoliosis, diagnosis or signs and symptoms of a disc herniation including pain or paresthesia below the knee [29, 30], history of spinal surgery or spinal fracture, systemic inflammatory, neurological, or other serious medical condition, or primary hip problem. People were also excluded if they were pregnant, receiving worker's compensation or disability benefits, in litigation for their low back pain problem, referred from a pain clinic, or presented with magnified symptom behavior [31]. The protocol for the clinical trial was approved by the university's Human Research Protection Office.

### 2.2. Laboratory Procedures

Subjects reported to the laboratory for pre- and posttreatment testing, approximately one week prior to initiation of treatment and one week following termination of treatment. At the pretreatment laboratory session, subjects provided written informed consent for participation in the study. They then completed several self-report measures including a demographic and low back pain history questionnaire [32], a numeric rating scale of symptoms for their low back pain during the previous week [33, 34], the modified Oswestry Low Back Pain Disability Questionnaire [35], and the Fear Avoidance Beliefs Questionnaire [36]. Next, subjects were classified into a low back pain subgroup according to the results of a standardized physical therapy examination based on the Movement System Impairment model for low back pain [5, 37]. Finally, kinematic data were collected for hip medial rotation, in addition to several other movement tests.

### 2.3. Kinematic Data Collection and Processing

**Figure 1:**
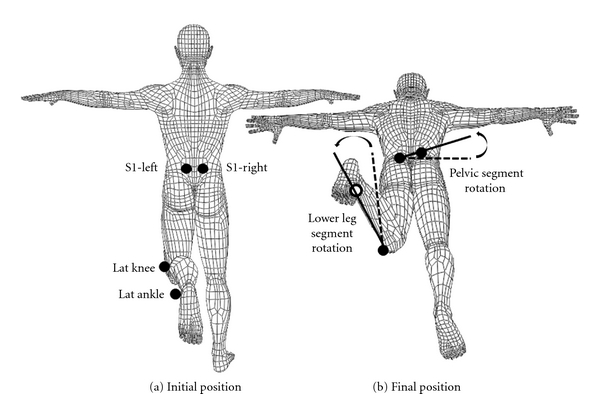
Hip medial rotation test and kinematic variables. (a) Posterior view of kinematic marker locations in the initial position: 7 cm left (S1-L) and right (S1-R) of the first sacral vertebra, on the lateral malleolus (lat ankle), and on the lateral knee joint line (lat knee). (b) Angled posterior view of the pelvic segment, defined by a line between the S1 markers, and the lower leg segment, defined by a line between the lat ankle and lat knee, in the final position of hip medial rotation. Lumbopelvic rotation was assessed by the angular excursion of the pelvic segment in the transverse plane between the initial and final positions. Hip medial rotation was assessed by the angular excursion of the lower leg segment in the transverse plane relative to the lumbopelvic rotation between the initial and final positions. Dotted lines indicate initial positions, and solid lines indicate final positions. Open circles indicate markers obscured from view.

A motion capture system with six cameras (EVaRT, Motion Analysis Corporation, Santa Rosa, CA, USA) was used for kinematic data collection and processing. Retroreflective markers placed 7 cm to the left and right of the first sacral vertebra (S1), on the lateral knee joint line, and distal to the lateral malleolus were used for the analysis of hip medial rotation (Figure 1(a)). A vector between the left and right S1 markers defined the pelvic segment, and a vector between the lateral knee joint line and the lateral malleolus markers defined the lower leg segment (Figure 1(b)). A sampling rate of 60 Hz was used to capture the data. For data collections, subjects were instructed to lie prone with one knee flexed to approximately 90 degrees and the hip in neutral rotation and adduction/abduction. Starting position was visually approximated by the testing physical therapist. Subjects were instructed to move the limb at a self-selected speed into hip medial rotation as far as they could and return to the starting position. They were allotted 10 seconds to complete this movement. No subjects exceeded this allotment, and, therefore, no subjects were instructed to perform the movement at a speed that was faster than what was natural for them. 

A fourth-order, dual-pass Butterworth filter with a cutoff frequency of 2.0 Hz was used to filter the data initially. Individual starts, ends, and movement times were calculated for the pelvic and lower leg segments. The start of motion for the pelvic segment was defined when its angular displacement exceeded 0.5° and its angular velocity exceeded 15% of its maximum. The end of motion for the pelvic segment was defined as the first point at which its angular position reached 99% of its maximum or the end of lower leg motion (see below), whichever occurred first. The start of motion for the lower leg segment was defined as the first point when its angular displacement exceeded 1.5° and its angular velocity exceeded 5% of its maximum. The end of lower leg motion was defined as the first point when its angular position reached 99% of its maximum. The parameters for the starts and ends of lumbopelvic and lower leg segment motion are similar to those used in studies of hip lateral rotation [12, 27]. Based on each subject's lumbopelvic segment movement time, the raw data were then filtered at a subject-specific cutoff frequency [38].

### 2.4. Treatment

Each subject received 6, 1-hour treatment sessions over a period of 6 weeks. Treatment included specific training to modify lumbopelvic motion during multiple exercises and functional activities in a manner consistent with their subgroup. All subjects in this analysis received specific training to limit lumbopelvic motion during hip medial rotation.

### 2.5. Dependent Variables and Data Analysis

Lumbopelvic rotation was represented by the angular displacement of the pelvic segment in the transverse plane. Total lumbopelvic rotation range of motion during hip medial rotation relative to the starting position was calculated. Hip medial rotation was calculated as the angular displacement of the lower leg segment relative to the rotation of the pelvic segment in the transverse plane (Figure 1). To understand how early or late in hip movement the lumbopelvic region began to move, the amount of hip medial rotation completed before the start of lumbopelvic rotation was calculated. Total hip medial rotation range of motion relative to the starting position was also calculated.

Values for total lumbopelvic rotation range of motion, hip medial rotation completed before the beginning of lumbopelvic rotation, and total hip medial rotation range of motion were compared between pre- and posttreatment laboratory sessions and between men and women. A 2 × 2 mixed model analysis of variance was used to analyze the data with time as the within-subjects factor and gender as the between-subjects factor. Alpha was set at 0.05.

## 3. Results

### 3.1. Subject Characteristics

Data for one male subject were removed from this analysis. At both the pre- and posttreatment laboratory testing sessions, he demonstrated lumbopelvic rotation range of motion greater than three standard deviations above the mean and was considered an outlier. There were no significant differences in any demographic or baseline low back pain variables between men and women, with the exception of height and weight (Table 1).

### 3.2. Lumbopelvic Rotation Range of Motion

Transverse plane lumbopelvic rotation range of motion means and standard deviations are presented in Table 2. There was not a significant interaction of gender and time on lumbopelvic rotation (*F *(1,29) = 2.08, *P* = 0.16, *η* ^2^ = 0.067). There was a significant main effect of time (*F *(1,29) = 34.01, *P* < 0.001; *η* ^2^ = 0.54). Both men and women decreased lumbopelvic rotation range of motion from pre- to posttreatment. There was also a significant main effect of gender (*F *(1,29) = 12.83, *P* = 0.001; *η* ^2^ = 0.31). Men demonstrated greater lumbopelvic rotation than women both pre- and posttreatment.

### 3.3. Amount of Hip Medial Rotation before the Start of Lumbopelvic Rotation

Lumbopelvic timing means and standard deviations are presented in Table 2. There was not a significant interaction of gender and time for the amount of hip medial rotation completed before the start of lumbopelvic motion (*F *(1,29) = 0.10, *P* = 0.76, *η* ^2^ = 0.003). There was a significant main effect of time (*F *(1,29) = 18.11, *P* < 0.001, *η* ^2^ = 0.38). Both men and women increased the amount of hip rotation completed prior to the onset of lumbopelvic rotation from pre- to posttreatment. There was also a significant main effect of gender (*F *(1,29) = 17.88, *P* < 0.001, *η* ^2^ = 0.38). At both pre- and posttreatment testing, men completed less hip rotation prior to the onset of lumbopelvic rotation compared to women.

### 3.4. Hip Medial Rotation Range of Motion

Hip medial rotation range of motion means and standard deviations are presented in Table 2. There was not a significant interaction of time and gender for active hip medial rotation range of motion (*F *(1,29) = 3.08, *P* = 0.091, *η* ^2^ = 0.10). Additionally, there was not a significant effect of time on active hip medial rotation range of motion (*F *(1,29) = 1.65, *P* = 0.21, *η* ^2^ = 0.05). Neither men nor women significantly changed their hip medial rotation range of motion from pre- to posttreatment. However, a significant gender effect indicated that at both pre- and posttreatment testing men demonstrated significantly less hip medial rotation compared to women (*F *(1,29) = 20.32, *P* < 0.01, *η* ^2^ = 0.41).

## 4. Discussion

Contrary to our hypothesis, women were no better at improving their lumbopelvic movement patterns during hip medial rotation than men. Both men and women decreased their lumbopelvic rotation range of motion and increased the amount of hip rotation completed before the start of lumbopelvic motion. Consistent with previous literature, men demonstrated greater lumbopelvic rotation that began earlier in the range of hip medial rotation compared to women before treatment [26]. Furthermore, results of this study demonstrate that baseline differences remain following treatment. These findings demonstrate that while men can improve lumbopelvic movement patterns with hip medial rotation, they still do not limit lumbopelvic motion during hip medial rotation to the level that women do.

Previous research suggests that people with low back pain may have more difficulty improving lumbopelvic movement patterns during a related movement, hip lateral rotation, than people without low back pain [17]. Potentially, greater lumbopelvic movement impairments observed during lower limb movements in people with low back pain [12, 17] make it harder for them to limit their lumbopelvic motion. Results of the current study, however, suggest that, in people with low back pain, greater baseline lumbopelvic motion during a lower limb movement may not interfere with the ability to improve with treatment. Despite displaying greater lumbopelvic movement impairments at baseline compared to women, men are also able to improve lumbopelvic movement pattern variables following treatment.

The results of the current study also suggest that classification-specific treatment based on the Movement System Impairment model effectively reduces lumbopelvic motion during hip medial rotation for both men and women. These results also suggest that men and women may be using similar strategies to reduce lumbopelvic motion during hip medial rotation. In particular, neither men nor women altered their hip medial rotation range of motion as a strategy to modify lumbopelvic motion. There was no change in hip medial rotation range of motion from before to after treatment for either gender. However, it is still important to note that men continued to demonstrate greater and earlier lumbopelvic motion than women, even after treatment. This suggests that men might need more emphasis or time spent on limiting lumbopelvic motion or even a modified treatment approach to reduce their lumbopelvic motion even further.

It is already known that, prior to treatment, men with low back pain demonstrate greater and earlier lumbopelvic rotation than women with low back pain during hip medial rotation [26]. Therefore, an alternative interpretation of the results of this study is that, following treatment, it is natural for men to display greater and earlier lumbopelvic motion than women. To our knowledge, no studies have examined the differences in lumbopelvic movement patterns during hip medial rotation between men and women without low back pain. So, it is unknown whether gender differences following treatment would parallel gender differences in a back-healthy population. The pattern men display could be due to physical factors that prevent them from being able to limit their lumbopelvic motion to the same level as women. Studies have demonstrated that both healthy men and men with low back pain have less active hip medial rotation range of motion than women [25, 26, 39]. Differences in the bony structure of the hip [40, 41] or in lower limb muscle characteristics [42–44] between men and women may account for differences in available hip medial rotation range of motion. Decreased available hip medial rotation range of motion in men compared to women may be driving increased and earlier lumbopelvic motion in men, even after they have learned to limit that motion to a certain extent. A different standard to judge lumbopelvic movement pattern impairments for men and women may be appropriate. The fact that men are able to improve may be more critical than the fact that they still display greater and earlier lumbopelvic motion than women after treatment. Future studies investigating lumbopelvic movement patterns in back-healthy men and women are necessary.

Repeated early and excessive lumbopelvic motion may contribute to low back pain by causing cumulative tissue stress, tissue damage, and pain [2, 5, 6]. Lumbopelvic motion during various movement, including hip rotation, has been linked to an increase in low back pain symptoms within a single session [14, 15]. Therefore, we believe that limiting lumbopelvic motion thought to be associated with symptoms is likely an important component of treatment for low back pain. Understanding how gender may affect a person's ability to limit lumbopelvic motion during certain limb movements is relevant to guide treatment in the clinical setting and for the design or interpretation of future research studies. However, we recognize that a limitation of this study is that we cannot directly determine whether limiting lumbopelvic motion during a single movement test, hip medial rotation, reduces low back pain symptoms for either gender. As participants in a larger clinical trial, the subjects in this study received a variety of other specific exercises and functional training, in addition to hip medial rotation. Posttreatment symptom behavior is likely the result of all the components of treatment and not only of learning to limit lumbopelvic motion during hip medial rotation. Results of the larger randomized clinical trial will better elucidate if limiting lumbopelvic motion as an underlying treatment principle is effective for reducing low back pain symptoms.

## 5. Conclusion

The results of this study demonstrate that while both men and women are able to limit lumbopelvic motion during hip medial rotation, men still demonstrate greater and earlier lumbopelvic motion than women after treatment. These results may have important treatment implications. They suggest that either (1) men need additional training to reduce their lumbopelvic motion to a level comparable to women or (2) a different standard should be used to judge lumbopelvic movement pattern impairments for men and women after treatment. To better understand these alternatives, future investigations are necessary to examine changes in symptom behavior in response to changes in lumbopelvic movement patterns during hip medial rotation for each gender.

## Figures and Tables

**Table 1:** Means (standard deviations) for subject characteristics.

	Male *N* = 15	Female *N* = 16
Height (cm)	178.47 (10.80)	163.49 (5.98)
Weight (kg)	81.85 (14.03)	63.56 (8.87)
BMI (kg/m^2^)	25.49 (2.06)	23.78 (3.19)
Modified Oswestry	21.47 (7.84)	23.25 (9.77)
FABQ-PA	12.93 (5.60)	13.13 (4.67)
FABQ-W	12.00 (10.97)	12.69 (4.99)
Time since onset of low back pain (years)	12.00 (8.74)	10.63 (8.96)
Number of flare-ups^a^ per year	6.14 (5.43)	5.77 (4.62)
Pain/paresthesia into thigh, *N* (%)	2 (13.3%)	3 (18.8%)
Current pain	3.43 (1.79)	3.03 (1.87)
Average pain^b^	4.13 (1.58)	3.72 (1.30)

^a^Von Korff, [28].

^b^Average pain over the past 7 days.

Modified Oswestry: Modified Oswestry Low Back Pain Disability Questionnaire, FABQ: Fear Avoidance Beliefs Questionnaire, PA: physical activity subscale, and W: work subscale.

**Table 2:** Means (standard deviations) for transverse plane lumbopelvic and hip medial rotation movement pattern variables.

	Lumbopelvic rotation ROM	Hip medial rotation before start of lumbopelvic rotation	Hip medial rotation ROM
Male			
Pretreatment	9.43 (4.54)	4.08 (2.68)	25.15 (6.61)
Posttreatment	3.62 (2.37)	11.81 (8.81)	25.61 (6.39)
Change (post-pre)	−5.81 (5.09)	7.73 (7.80)	0.47 (4.90)
Female			
Pretreatment	5.16 (3.57)	12.77 (7.89)	40.30 (10.53)
Posttreatment	1.65 (2.14)	21.74 (10.91)	37.28 (10.23)
Change (post-pre)	−3.51 (3.75)	8.97 (13.18)	−3.02 (6.04)

ROM: range of motion.
